# Kinetics of the invasion and egress processes of *Babesia divergens*, observed by time-lapse video microscopy

**DOI:** 10.1038/s41598-018-32349-7

**Published:** 2018-09-20

**Authors:** Elena Sevilla, Luis Miguel González, Daniel Luque, Jeremy Gray, Estrella Montero

**Affiliations:** 1 Parasitology Department, Centro Nacional de Microbiología, Instituto de Salud Carlos III, Madrid, 28222 Spain; 2 Electronic and Confocal Microscopy Department, Unidades Comunes Científico-Técnicas (SG-SAFI), Instituto de Salud Carlos III, Madrid, 28222 Spain; 3 UCD School of Biology and Environmental Science, University College Dublin, Dublin 4, Ireland

## Abstract

Based on confocal fluorescence and bright field video microscopy, we present detailed observations on the processes of invasion and egress of erythrocytes by the apicomplexan parasite *Babesia divergens*. Time-lapse images reveal numerous unexpected findings associated with the dynamics of *B. divergens* and its ability to manipulate the erythrocyte during both processes in its asexual cycle under *in vitro* conditions. Despite the speed at which these processes occur and the small size of the parasite, we capture infective merozoites moving vigorously and causing striking deformations in the erythrocyte’s plasma membrane during an active invasion. We also observed intraerythrocytic dynamic stages as paired pyriforms, double paired pyriforms, tetrads, unattached pyriform sister cells and multiple parasite stages resulting in the release of large numbers of merozoites over a short period. Of considerable interest is that time-lapse images reveal a novel mechanism of egress used by *B. divergens* to exit the human erythrocyte. The release occurs when *B. divergens* parasites establish contacts with the plasma membrane of the erythrocyte from within, before exiting the cell. Visualization and analysis of the images enabled us to obtain useful information and broaden our knowledge of complex and crucial events involved with parasitisation of human erythrocytes by *B. divergens*.

## Introduction

*Babesia divergens* is an apicomplexan protozoan parasite that is naturally transmitted by ixodid ticks^1^. *B. divergens* cases are concentrated in Europe where the intraerythrocytic parasite is considered highly pathogenic to cattle, in which it causes babesiosis, commonly known as redwater^2,3^. This parasite also causes severe babesiosis in humans, occurring as a fulminant infection. *B. divergens* infections are therefore considered medical emergencies and patients require immediate treatment^1,4^.

Once the host has been bitten by an infected tick, *B. divergens* sporozoites invade the erythrocytes, and the asexual cycle of the parasite then commences. The asexual multiplication of *B. divergens*, is asynchronous^5^ and parasites multiply in erythrocytes by binary fission, resulting in a considerably complex pleomorphic process^2^. Recently, it has been possible to elucidate a temporal and coordinated proliferative cycle *in vitro* over 24 hours^6^. This 24 h cycle comprises seven intraerythrocytic parasite “stages”*^1^^6^ (that are not necessarily sequential) namely: single round trophozoite, double trophozoites (two round unattached cells), quadruple trophozoites (four round unattached cells), paired pyriforms (two attached pear-shaped sister cells), double paired pyriforms (two sets of paired sister cells), tetrads or Maltese Crosses (four attached sister cells), and multiple parasites (erythrocytes containing more than four parasites). Double or more unattached parasites are capable of exiting at any time and the released merozoites invade new erythrocytes. As a result, cultures, initially synchronous, become increasingly asynchronous after 24 hours and gain complexity and heterogeneity, mimicking the situation in human infections^6^.

Egress and invasion are critical processes of the *B. divergens* life cycle in which the merozoite is vulnerable and therefore an excellent therapeutic target.

Some features of the invasion process, observed by optical and transmission electron microscopy (TEM), suggest that *B. divergens* invasion may occur rapidly, within 45 to 60 s^5,7,8^. The free merozoite of *B. divergens* recognizes the erythrocyte through its apical end and causes an initial local depression in the host cell membrane, which deepens with parasite invasion^9^. A dynamic tight junction is formed between the free merozoite membrane and the plasma membrane of the erythrocyte^7–9^. This connection, thoroughly studied in *Plasmodium falciparum*^10–12^, moves across the merozoite surface from anterior to posterior during the entry of the parasite and is essential for crucial invasion events. Invasion is also accompanied by release of the contents of the apical organelles. Rhoptries, dense granules and micronemes, are present in the free merozoite apical end of *B. divergens* suggesting that these organelles secrete the parasite ligands at the initiation and duration of invasion^7–9,13,14^. Internalization of *B. divergens* merozoites occurs through the formation of a parasitophorous vacuole (PV) during invagination of the erythrocyte membrane^7,9,15^. Then *B. divergens* dissociates from the PV to make direct contact with the cytoplasm of the erythrocyte^15^.

Despite knowledge of some features of the *B. divergens* asexual life cycle, essential processes remain unknown or are not yet entirely understood. In this paper, we focus on real-time imaging of the morphological steps and kinetics of live *B. divergens* parasites during the invasion and egress processes by exploiting time-lapse videomicroscopy, confocal fluorescence and bright field imaging. Our data reveal the kinetics of the erythrocyte invasion process conducted by vigorous free merozoites and an unexpected active egress process of dynamic intraerythrocytic stages from the infected erythrocyte.

## Results

### Toxicity of labels for parasites and erythrocyte plasma membranes

The red fluorescent lipid analogue PKH26 and the fluorescent dye MitoTracker green, which accumulates in active mitochondria, were used to label, respectively, the plasma membrane of the human erythrocytes and the mitochondria of parasites present in *B. divergens* asynchronous cultures. Then, the toxicity of PKH26 and MitoTracker green was evaluated simultaneously by monitoring the viability and growing of double-stained cultures. Non-stained culture controls were used in parallel. All culture samples, with an initial parasitemia of 19.2% were incubated under growing conditions. Twenty-four hours post-incubation, cultures were stained with Giemsa and observed by light microscopy. Non-stained and double-stained cultures progressed adequately and parasitemia increased up to 28.5% ± 0.2 (mean ± standard deviation) and 23.9% ± 0.3 respectively. Double-stained fluorescent parasites and erythrocytes retained their morphology and no crisis forms within the erythrocytes were observed by light microscopy. Moreover, red fluorescent erythrocytes and green fluorescent parasites were observed immediately after staining and 24 hours later under the fluorescent confocal microscope, confirming that both parasites and erythrocytes remained viable and labeled according to the time-lapse images and videos recorded.

### Time-lapse microscopy of *B. divergens* live parasites

**Figure 1:**
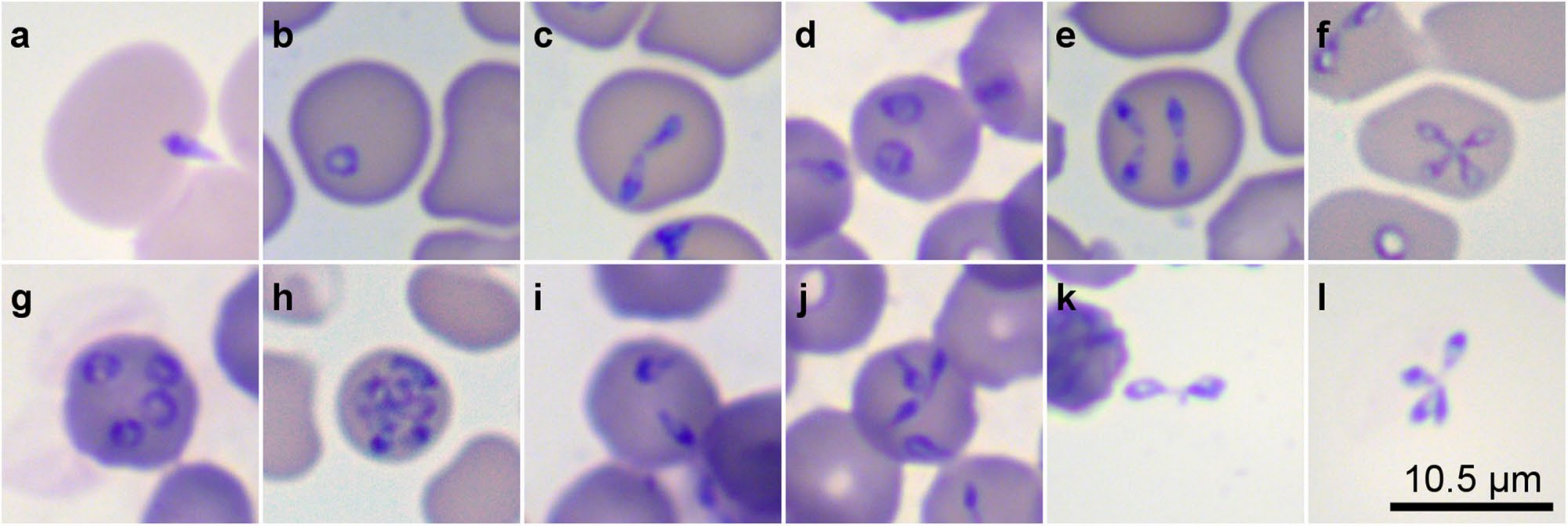
*B. divergens* forms inside and outside erythrocytes were identified in *in vitro* cultures by Giemsa staining. (**a**) A free merozoite invading a human erythrocyte. Panels b–j show different stages within the human erythrocyte. (**b**) Single round trophozoite. (**c**) Paired pyriforms, a stage formed by two attached pear-shaped sister cells. (**d**) Double round trophozoites, (**e**) Double paired pyriforms. (**f**) Tetrad (**g**). Quadruple round trophozoites. (**h**) Multiple parasites. (**i**) Double unattached pyriforms. (**j**) Quadruple unattached pyriforms. (**k**) Intact paired pyriform outside the erythrocyte. (**l**) Intact tetrad outside the erythrocyte. Slides were examined with a Primo Star microscope (Zeiss, Germany) at 100X magnification.

We used time-lapse microscopy and double-labeled cultures immediately after staining and 19 hours later to film several dynamic free merozoites (Fig. 1a) searching for a suitable erythrocyte to invade, and 11 successful invasion events. Video data also revealed the dynamic egress process of 265 infected erythrocytes showing identifiable *B. divergens* live stages within. Of the total events captured, 88 stages (33.2%) corresponded to paired pyriforms (Fig. 1c). Seventy-one stages (26.8%) corresponded to double paired pyriforms (Fig. 1e). Twenty-three of the total (8.7%) were tetrads (Fig. 1f) and 76 were multiple parasites stages (28.7%), (Fig. 1h). Eventually, we observed the egress process of double (1.1%, n = 3) and quadruple (1.5%, n = 4) unattached pyriform parasites (Fig. 1i,j) within the erythrocyte.

### Searching behavior and recognition of erythrocytes by free merozoites

We observed elongated pyriform free merozoites, with a polarized morphology, moving vigorously in all directions and covering distances much larger than their own length (≈ 3 µm)^9^ searching for a suitable erythrocyte to invade. Free merozoites moved in all directions at an average speed of ≈3 µm/s with a complex motility. The paths they took were straight, circular, zigzagging, back and forth or even up and down (Supplementary Video S1). However, we did not clearly discern twirling or helicoidal movements, such as has been observed for *T. gondii*^16^ and *B. bovis*^17^ respectively.

The free merozoite searched for and recognized the host cell causing a deep depression in the erythrocyte plasma membrane. The recognition step between the merozoite and the erythrocyte sometimes appeared to be reversible, because in a small number of cases the free merozoites that made contact with the cells immediately separated to search for other candidates. In fact, merozoites during its trajectory dragged, flipped and rotated many erythrocytes, reducing its speed on contact with erythrocytes but recovering its movement and speed when resuming their trajectories (Supplementary Video S1). Other merozoites remained firmly attached to the erythrocyte, suggesting the formation of irreversible tight connections. Despite the degree of the erythrocyte deformation and the tight binding caused by free merozoites, a successful invasion process was not always ensured. In fact, many observed merozoites were unable to invade human erythrocytes.

### The invasion process of *B. divergens* free merozoites

**Figure 2:**
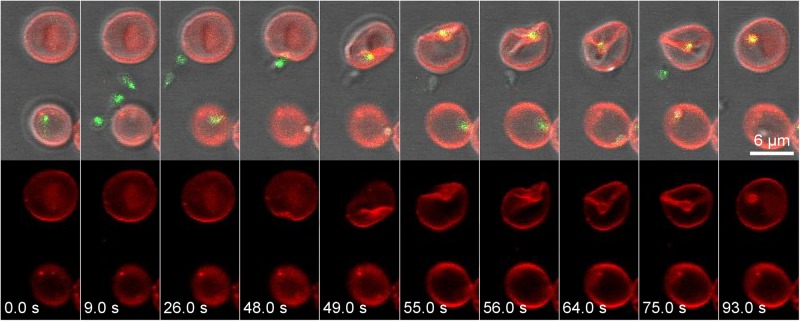
The *B. divergens* free-merozoite recognizes and invades the human erythrocyte. The panels above show time-lapse image sequences of four *B. divergens* merozoites stained with MitoTracker (green fluorescent) that are exiting in 9 s from an infected human erythrocyte stained with PKH26 (red fluorescent). One of the merozoites identifies a new erythrocyte and establishes a tight irreversible connection with the erythrocyte plasma membrane causing a deep invagination and deformation to the erythrocyte at 48–75 s. The total internalization of the free merozoite occurs at ≈93 s. The parasite is surrounded by the red fluorescent erythrocyte membrane-derived parasitophorous vacuolar membrane after invasion. Simultaneously, the panels below show the PKH26 fluorescence around the infected and non-infected erythrocytes but also around the invasive parasite within the erythrocyte. Time-lapse imaging was captured every 1 s. The time-lapse between each frame is indicated in seconds.

We observed 11 free merozoites that successfully completed the invasion process. The free merozoite attacked the erythrocyte and caused a dramatic depression in the plasma membrane, deforming the cell. Surprisingly, the wide apical end of the merozoite^9^ was covered and surrounded by the membrane by curving and forming deep folds in the erythrocyte plasma membrane (Fig. 2). When an interaction was firmly established between the merozoite and the erythrocyte the so-called pre-invasion phase^18^ finished and a second penetrative phase commenced. In this phase of invasion the merozoite entered the erythrocyte. Simultaneously, the PV was formed and appeared as a red fluorescent membrane structure around the green fluorescent parasite (Fig. 2). At the end of the process the parasite fully entered the erythrocyte and remained immobile inside the PV changing immediately from pyriform to a round shape. The erythrocyte returned to its normal shape and size a few seconds later (Supplementary Video S2).

The invasion time recorded for each free merozoite was different and was mainly influenced by the time for the pre-invasion phase. The invasion process was completed on average in 72.2 s ± 27.02 and the fastest and longest time taken for merozoites to invade the erythrocyte was 38 s and 126 s respectively. The pre-invasion phase time was estimated from the release of the free merozoite to the establishment of the tight connection with the erythrocyte plasma membrane and took on average 41.7 s ± 25.47. The internalization of the free merozoite during the invasive phase took on average 34.3 s ± 12.85.

Exceptionally, time-lapse images showed one erythrocyte attacked simultaneously by two merozoites. Only one was seen clearly invading the cell, which took 38 s, while the second merozoite remained fixed to the outside of the erythrocyte plasma membrane. In fact, merozoites were not observed to invade cells that were already parasitised.

### The egress process of *B. divergens* parasites from the human erythrocyte

**Figure 3:**
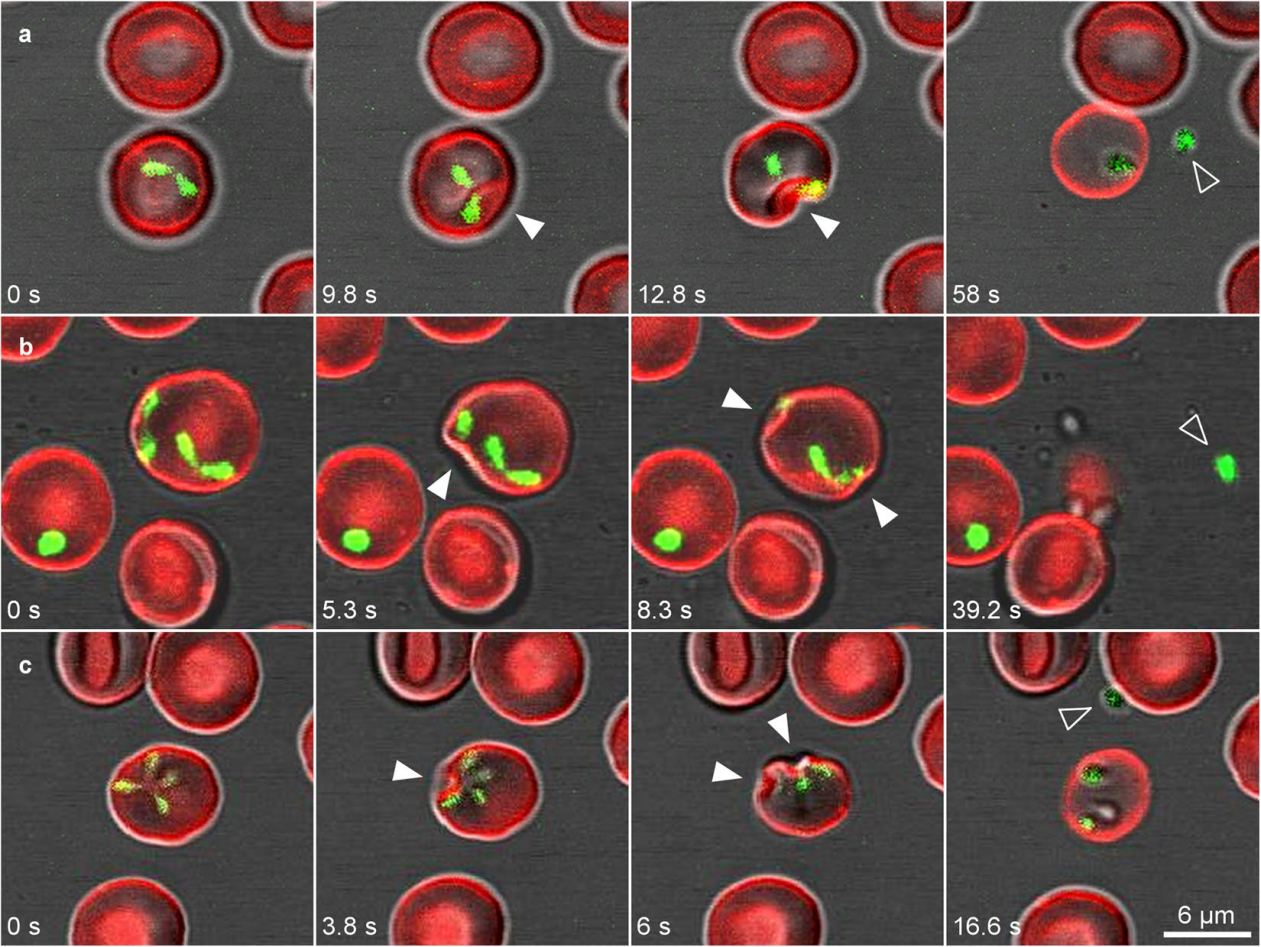
*B. divergens* intraerythrocytic stages use similar strategies to exit from the infected erythrocyte. Time-lapse image sequences of the egress process of *B. divergens* parasites stained with MitoTracker (green fluorescent) from infected erythrocytes stained with PKH26 (red fluorescent). (**a**) A merozoite appears to emanate after the dramatic invagination (white arrowheads) of the erythrocyte plasma membrane provoked by the paired pyriform at 9.8–12.8 s. Seconds later, one free merozoite (arrowhead) is released from the erythrocyte while the other one remains within the host cell. (**b**) Egress process of a double paired pyriform. Both paired pyriforms produce successive dramatic depressions (white arrowheads) of the erythrocyte plasma membrane after 5.3 s and 8.3 s causing the discharge of one free merozoite (arrowhead) and the deformation of the erythrocyte. The other three merozoites are retained within or trapped outside the erythrocyte. (**c**) A tetrad causes an initial invagination in the erythrocyte plasma membrane (white arrowheads) at 3.8 sec. The depression and deformation of the erythrocyte become even clearer at 6 s. The tetrad can dissociate into four merozoites inside the cell. Only one free merozoite (arrowhead) is being released from the infected erythrocyte. Time-lapse imaging was captured every 0.753 s. The time-lapsed between each frame is indicated in seconds.

Time-lapse imaging showed different dynamic stages moving within the erythrocyte and preparing for an active egress process. It is noteworthy that paired pyriforms, double paired pyriforms, tetrads, multiple parasites stage and double and quadruple unattached pyriform parasites shared some similar features during the exit. These stages, before exiting the cell, established contacts with the plasma membrane of the erythrocyte from within and produced a dramatic depression of this plasma membrane (Fig. 3a–c; Supplementary Video S3). The invagination and deformation of the erythrocyte plasma membrane occurred very rapidly, in less than a second, and it was not always possible to observe this phenomenon during egress. Then, paired pyriforms (Fig. 3a), double paired pyriforms (Fig. 3b) and tetrads (Fig. 3c) usually detached rapidly and became merozoites. Occasionally, a few tetrads detached first and the resulting merozoites established contacts with the erythrocyte before egressing.

In general, while some of the resulting merozoites were released immediately from the erythrocytes as invasive parasites or free merozoites, others failed to exit and were retained inside the erythrocyte or became trapped by the outside erythrocyte plasma membrane (Supplementary Videos S3–S5).

**Figure 4:**
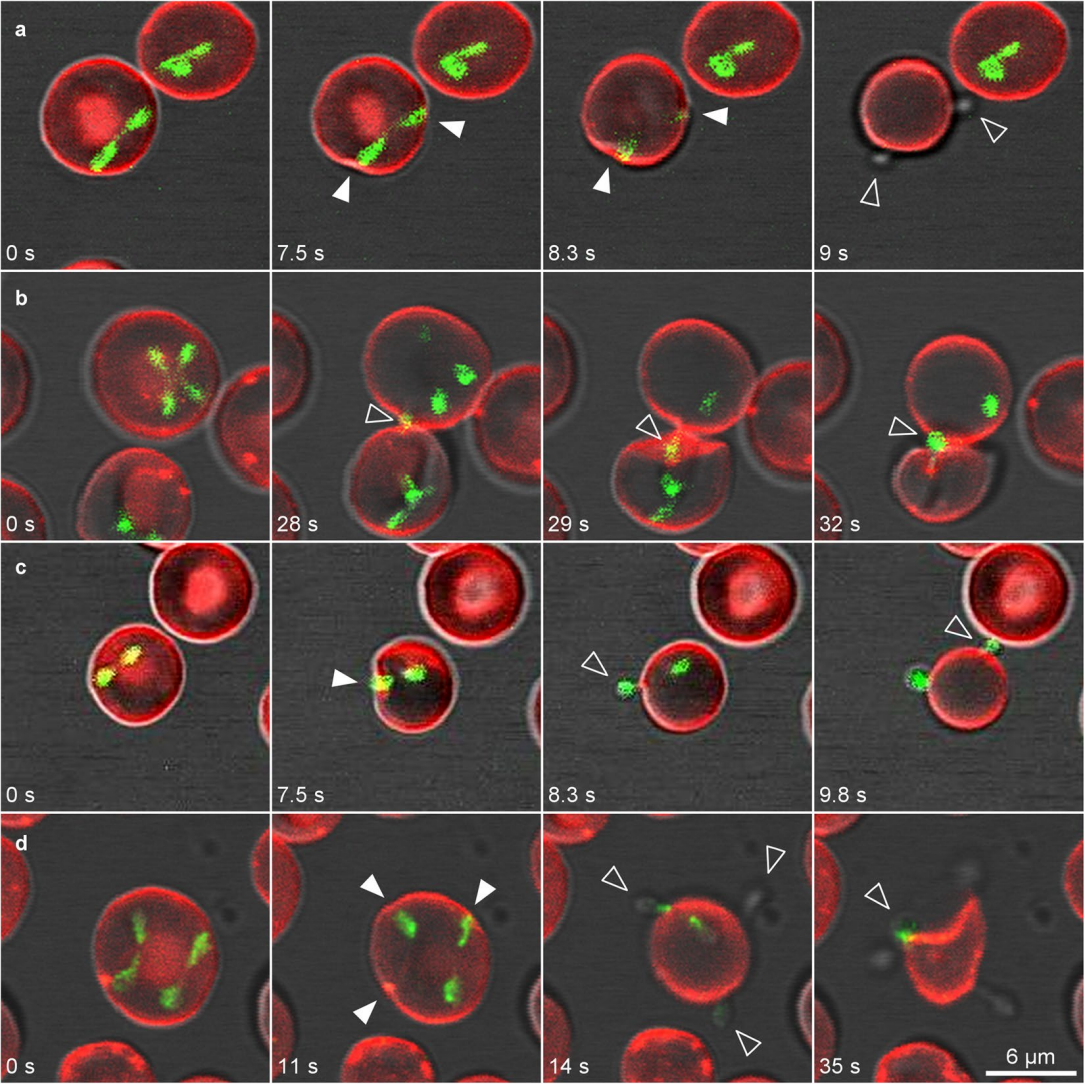
*B. divergens* merozoites exit the erythrocyte. Time-lapse image sequences of *B. divergens* parasites stained with MitoTracker (green fluorescent) and erythrocytes stained with PKH26 (red fluorescent). (**a**) A paired pyriform produces simultaneously depressions (white arrowheads) on two opposite sides of the erythrocyte plasma membrane at 7.5–8.3 s. Then, the two sister cells comprising the paired pyriform exert enough force in opposite directions to detach from each other and the detachment process results in two merozoites (arrowheads) in 8.3 s. Finally, both merozoites are simultaneously released from the opposite sides of the infected erythrocytes in 9 s. (**b**) An unattached merozoite from a tetrad touches the erythrocyte plasma membrane and is released (arrowhead) in 28 s following by two merozoites (arrowheads) that leave the cell from the same side in 29 s and 32 s, respectively. One merozoite remains within the erythrocyte. (**c**) Erythrocyte plasma membrane invagination (white arrowheads), causing by a paired pyriform at 7.5 s, facilitates the release of the two merozoites (arrowheads) from opposite sites. The first merozoite is released from the erythrocyte in 8.3 s and the second one in 9.8 s. (**d**) Two paired pyriforms (double paired pyriforms) simultaneously produces depressions on three different sides (white arrowheads) of the erythrocyte plasma membrane at 11 s. Then, four merozoites (arrowheads) are released from different sides at 14–35 s from the infected erythrocyte. Time-lapse imaging was captured every 0.753 or 1 s. The time-lapsed between each frame is indicated in seconds.

Two or more merozoites that exited erythrocytes, were released simultaneously from different sides (Fig. 4a; Supplementary Video S6) or were released one after each other. In this latter case, merozoites were released from the same side (Fig. 4b; Supplementary Video S7), from opposite sides (Fig. 4c; Supplementary Video S8) or from different parts of the plasma membrane (Fig. 4d; Supplementary Video S9). Hence, different parts of the erythrocyte membrane were selected for exit by different parasites.

In the particular case of multiple parasites stages, formed by complex and different combinations of paired pyriforms, tetrads and unattached parasites within the same infected erythrocyte, the majority of parasites exited rapidly and almost simultaneously, but some were left behind (Supplementary Video S10).

It is noteworthy that, paired pyriform was able to exit while the sister cells were still attached to each other from erythrocytes infected with paired pyriforms, double paired pyriforms, tetrads or multiple parasites. After egress, the paired pyriforms became trapped by the outside erythrocyte membrane (Supplementary Video S11), remained attached outside the erythrocyte (Supplementary Video S10) or detached from each other to yielded two free merozoites (Supplementary Video S12). Paired pyriforms outside the cell moved slowly and remained next to the abandoned erythrocyte. In fact, we did not observe these forms invading new erythrocytes.

Intact tetrads were able to exit as well. The evaluation of *B. divergens* cultures, stained with Giemsa by using light microcopy, additionally confirmed the presence of these well-preserved entire intact forms outside erythrocytes (Fig. 1k,l). Eventually, we observed double and quadruple unattached pyriform parasites (Fig. 1i,j). These stages, which were capable of exiting the erythrocyte (Supplementary Video S13), have an unclear origin and are not included in the 24 hour-*in vitro* cycle model proposed for *B. divergens*^6^.

The egress process of the intraerythrocytic parasites was usually fast. Most of the egress events occurred in less than 30 s. The average time taken for a paired pyriform to exit the cell was 23.2 s. The fastest time recorded was 1.3 s and the longest 101 s. The egress process of double and quadruple unattached pyriform parasites occurred on average in 25.3 s and 26.7 s respectively. The fastest and longest times for double unattached pyriform sister cells were 11 s and 42 s and for quadruple unattached pyriform sister cells 10 and 63.9 s respectively. Multiple parasite egress occurred in an average of 28 s, the fastest and longest egress time recorded being 1 and 125 s respectively. Double paired pyriform and tetrad stages took more time than other stages to exit −41.9 and 39.8 s respectively (P = 0.0143). The fastest times were 2 and 6 s respectively and the longest times were 148 and 159 s respectively.

**Figure 5:**
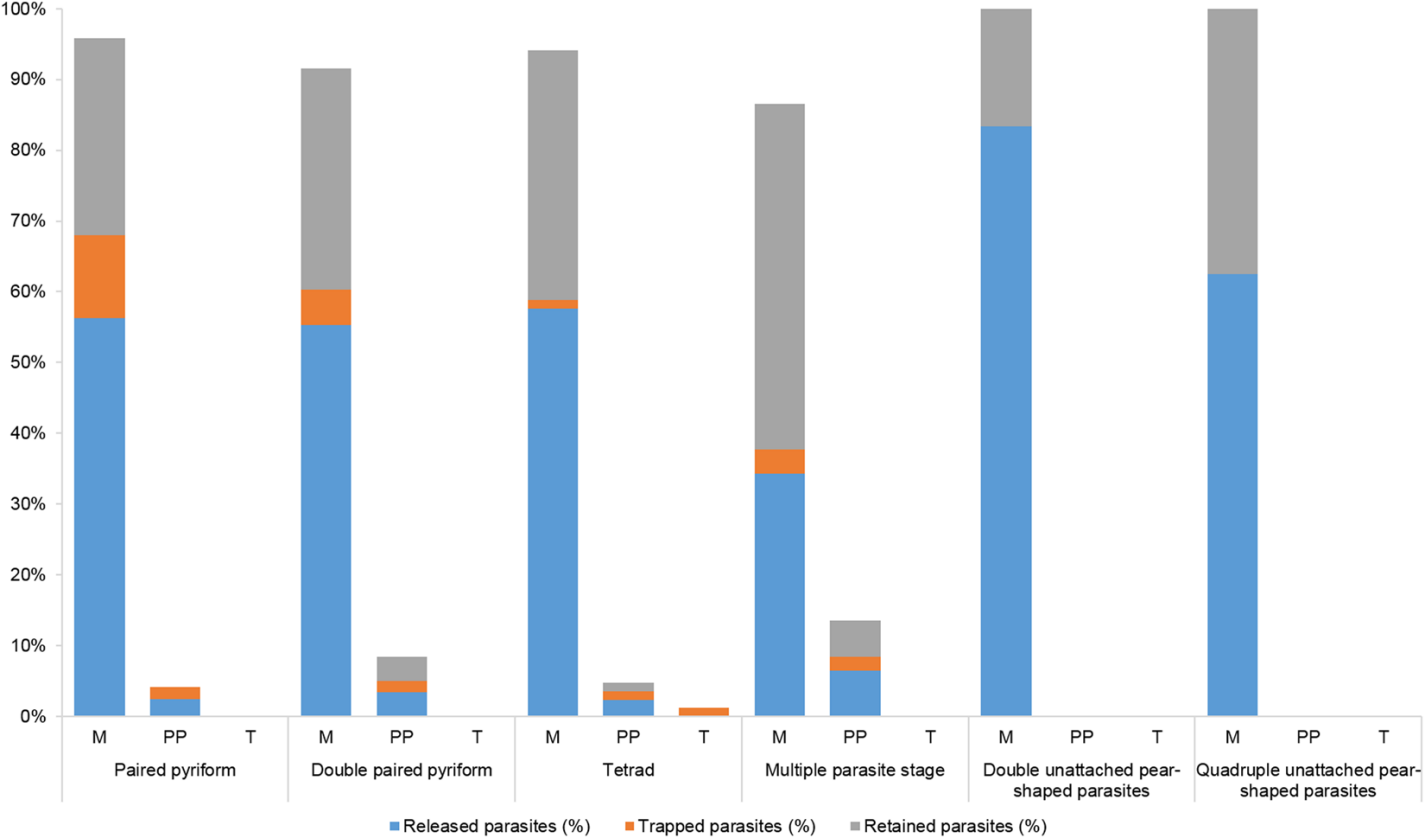
The egress efficiency of *B. divergens* was determined by the rate of parasite yield by the intraerythrocytic stages during the process. The graph shows the percentage of released, trapped and retained parasites within the infected erythrocytes: merozoite (M), paired pyriforms (PP), tetrads (T), n values were: n = 88 paired pyriforms, n = 71 double paired pyriforms, n = 23 tetrads, n = 76 multiple parasites stages, n = 3 double unattached pyriform parasites, n = 4 quadruple unattached pyriform parasites.

To assess the efficiency of the intraerythrocytic stages in exiting the erythrocyte, we counted the number of released parasites, the parasites that became trapped by the outside erythrocyte membrane and the parasites that remained inside the erythrocyte during each egress process (Fig. 5).

### Erythrocyte damage caused by parasite egress

The erythrocyte underwent an invagination of its membrane and sometimes the cell was contracted while parasites were released. Both events caused morphological changes and a decrease of the optical density and diameter of the infected erythrocyte during the egress process (Supplementary Video S2). The average of the initial diameter of the infected erythrocytes was 8.42 µω ± 0.78 (n = 265). Non-infected erythrocytes showed a similar size of 8.21 µm ± 0.72 (n = 265). We did not observe any significant correlation in the size of the diameter of the human erythrocyte in relation to the stage or the number of parasites within the erythrocyte before egress. During and after egress, the diameter of infected erythrocytes rapidly decreased by 1.13 µm ± 0.6. The average of the final diameter was 7.29 ± 0.63 µm. (P < 0.0001).

## Discussion

Invasion and egress of *B. divergens* are complex processes determined by the different stages that are outside and inside the infected erythrocyte. Numerous steps are completed during entry and egress of *B. divergens* and each of the events observed takes place at high speed; therefore, they are not always visible at the image-capture intervals we used. Moreover, it should be noted that this study was conducted using *in vitro* conditions that do not reflect the complexity of an *in vivo* environment. Thus, some observed aspects could differ from the *in vivo* situation. Nevertheless, time-lapse videomicroscopy enabled us to provide a detailed description of the processes that are essential for the survival of *B. divergens*.

Both inside and outside the erythrocyte, the parasite makes vigorous contact with the host cell, and in particular its behavior during the invasion process is similar to that of other apicomplexan parasites, such as *Plasmodium falciparum*, *P. yoelii*, and *B. bovis*, which also invade erythrocytes^17–19^.

*B. divergens* free merozoites cover distances from ≈10 µm to more than 100 µm until erythrocyte contact and invasion occur. It has been previously reported that the first contact between an invasive *Babesia* merozoite and an erythrocyte is random^9^, however time-lapse images recorded could not capture this specific event, nor reorientation of the merozoite to contact the erythrocyte membrane with the apical end.

**Figure 6:**
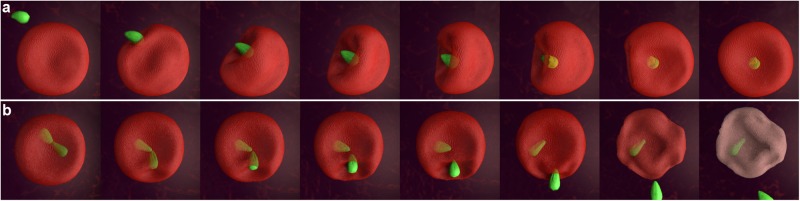
Serial diagram showing the most representative steps of the invasion and egress process of *B. divergens*. (**a**) The merozoite contact with the erythrocyte is vigorous and cause a deep invagination and waves of deformation on the erythrocyte membrane plasma during the entry. (**b**) Serial events represent a novel mechanism of egress used by *B. divergens* to exit the human erythrocyte. The intraerythrocytic parasite attaches to the erythrocyte to internalize the erythrocyte plasma membrane, a strategy that could be used by the parasites to exit the erythrocyte.

By its own movements, the free parasite generates sufficient force to manipulate erythrocytes energetically, displacing and deforming them. The erythrocyte in turn is sufficiently flexible to fold in on itself while undergoing attack and invasion by the parasite (Fig. 6a; Supplementary Video S14). The time taken by the merozoite to invade and the degree of deformity it generates in the erythrocyte membrane varies in each event. This fact could be associated with the time taken by each merozoite to relocate itself and establish firm contacts and efficient molecular interaction until it manages to enter. However, contact with the erythrocyte plasma membrane does not guarantee that all the merozoites enter. Thus, in our study, the merozoites that invaded the cell did so in ≈2 minutes, after which time the merozoites that did not enter remained attached to the erythrocyte plasma membrane, although they had lost their ability to invade.

Free merozoites were not observed to invade cells that were already parasitized. This phenomenon has been associated recently with a possible electrical mechanism of repulsion that could prevent free *Babesia* merozoites from interacting with already infected erythrocytes^20^. This fact also suggests that the polyparasitism observed in multiple parasites stages probably results from continuous cycles of division from an original invading parasite rather than by multiple infections of the same erythrocyte. Notably, tetrads and polyparasitism seem to be infrequent in the bovine host *in vivo* but are commonest in human and gerbil highly parasitized infections^3,21^, where normal controls of parasite propagation^6^ could be compromised, preventing the regular release of merozoites.

For the parasite egress process, time-lapse video microscopy showed dynamic paired pyriforms, double paired pyriforms, tetrads, double and quadruple unattached pyriform cells and multi-parasite stages forming a reservoir of parasites capable of exiting the infected erythrocyte in the form of free merozoites to invade new erythrocytes.

During egress, intraerythrocytic parasites come into contact with the erythrocyte from the interior, thus leading to internalization of the erythrocyte plasma membrane to the cytoplasm (Fig. 6b; Supplementary Video S15). This strategy probably facilitates the expulsion of free merozoites and, to a lesser extent, intact attached forms that, once outside the cell, either remain attached together or separate as free merozoites. The egress of these intact forms (paired pyriforms and tetrads) was not observed in a recent study of a 24 hour-synchronized cycle of *B. divergens*^6^. Neither double or quadruple unattached pyriform parasites were included in their observations. Hence, this phenomenon could occur specifically in highly parasitized asynchronous cultures and could be determined by the complex nature of the replication and propagation of the parasite *in vitro*.

Dissociation of single paired pyriforms and tetrads into 2 or 4 sister cells that immediately exit the erythrocyte, is yet another point of interest in the process of egress. The videos show how the attached sister cells generate opposing traction forces of such magnitude that they manage to separate from one another mechanically^22^ along their narrowest part in a few seconds. Once fission is complete, the resulting merozoites do not appear to have been damaged at their posterior end, since they can move freely inside and outside the infected erythrocyte.

Independently of the total number of events observed, the graph in Fig. 5 shows the yield of each stage during egress. The paired pyriforms, double paired pyriforms, and tetrads can release more than 50% of the parasite load in the form of free merozoites and a somewhat lower proportion in the form of paired pyriforms. A small percentage of merozoites, paired pyriforms, and tetrads remain attached and trapped on the outside of the erythrocyte. In each of these 3 cases, we observed that the parasite does not invade new cells. Of note, the percentage of parasites that are not released during egress of single paired pyriforms, double paired pyriforms, and tetrads is very similar between the stages, with 27.8%, 34.7%, and 36.5% of the parasite load, respectively, inside the infected erythrocytes. In the case of the erythrocytes harboring numerous multiple parasites, the yield is inverted with respect to the three previous stages, and they release 34.3% of their parasite load to the medium in the form of free merozoites and 6.4% in the form of single paired pyriforms. However, 54% of the load is retained inside the infected erythrocyte. The double and quadruple unattached pyriform sister cells were more efficient and released 83.3% and 62.5% of their merozoites, respectively, during egress (Fig. 5). These results could indicate that egress is more efficient in erythrocytes infected with 2 or 4 attached or unattached parasites than in erythrocytes infected with more than 4 individuals (P < 0.05). In this latter case, egress could be less efficient because the large number of parasites trying to exit the cell hamper each other’s progress both within the infected erythrocyte and during egress. In addition, some of the parasites that do not exit the erythrocyte could be physiologically dysfunctional.

The egress process does not always guarantee the release of 100% of the parasite load; however, it probably causes irreversible damage to the erythrocyte. The infected erythrocyte, which is in suspension, is not destroyed outright by egress but loses its tensed spherical shape and morphology during and after the process and is reduced in optical density and size. This may be one of the reasons why escape of some parasites is hampered and others remain attached to the external surface of the erythrocyte plasma membrane instead of being released. In similar observations, high-speed DIC video microscopy provided accurate images showing that egress of *P. falciparum* occurs sequentially, with curling and eversion of the erythrocyte membrane necessary for merozoites to disperse and avoid being trapped^23^.

Figure 6 recreates how erythrocyte density decreases as a consequence of the egress process, a phenomenon that is clearly observed in the bright field channel of the Supplementary Video S2. We speculate that the plasmatic membrane rupture of the infected erythrocyte could be accompanied by hemoglobin release during and after the egress process. This fact could partly explained hemolysis, altered levels of bilirubin and jaundice in babesiosis^1–3^_._

Of considerable interest is the fate of the resulting damaged erythrocytes. Taking into account that the spleen removes older or damaged erythrocytes and leads to the efficient removal of blood-borne microorganisms^24,25^ such as *Babesia* spp^26^ and also cellular debris, the small erythrocytes that result from egress of *B. divergens*, are probably removed from the circulation by the spleen soon after parasite egress.

In conclusion, our study provides new data on the kinetics of invasion and egress of *B. divergens* and suggests that the deformations and invaginations that the plasma membrane undergoes as a consequence of the action of the parasite, constitute a commonly and central component of both processes.

In most cases during both entry and egress, the widest part of the merozoite where the apical complex of the parasite is located^9^, is that which comes into contact with the erythrocyte. This area of contact could be the enclave where ligands, proteases, and adhesins of the parasite^8,13,14,27^ and glycophorin receptors of the erythrocyte^28^ facilitate entry of the free merozoite.

Specifically, and according to what we observed, invasion could be brought about by the high degree of flexibility of the erythrocyte membrane on close contact with the free merozoite. These waves of deformation are associated with distortions in the erythrocyte cytoskeleton, which in the case of *P. falciparum* are mediated by the high Ca^2+^ levels generated on contact between the free merozoite and the erythrocyte membrane during invasion^29,30^. Stimulation of the ß-adrenergic receptors by *P. falciparum*^18^ of the erythrocyte plasma membrane^31,32^ also leads to an increase in cyclic AMP and deformation of the cell. Consequently, the area of contact between the parasite and the membrane increases, thus facilitating reorientation and entry.

Similarly, during the process of egress, it might seem that *B. divergens* attaches to the erythrocyte to internalize it, thus generating tension in the erythrocyte plasma membrane that could be used by the parasites to propel themselves outwards.

Future challenges and endeavours reside in understanding how and at what point molecular mechanisms associated with mechanical properties (e.g. force and motility of the parasite or flexibility of the erythrocyte) come together to facilitate critical processes necessary for *B. divergens* to propagate during its asexual life cycle.

## Materials and Methods

### Ethics statement

Human A+ blood from healthy volunteer donors was used to maintain blood stage cultures of *B. divergens*. The blood and protocol were approved for use by the Blood Transfusion Center in Madrid, Spain. All methods were performed in accordance with the relevant guidelines and regulations of the institution. Donors provided informed written consent for use of their blood for research purposes.

### Parasite propagation

The *B. divergens* asynchronous cultures (Bd Rouen 1986 strain) were maintained *in vitro* as previously described^21^ in human A+ erythrocytes at 5% hematocrit in complete medium (RPMI 1640 Gibco, Grand Island, NY, USA) supplemented with 10% human serum (The Interstate Companies, Memphis, TN), 7.5% (wt/vol) sodium bicarbonate solution (Lonza Group Ltd, Basel. Switzerland) and 50 µg/ml hypoxanthine (Sigma-Aldrich Corporation, St Louis, MO). Cells were cultured at 37 °C in a humidified atmosphere of 5% CO_2_.

### Staining *B. divergens* culture-parasites with PKH26 and MitoTracker green

PKH26 red fluorescent cell linker kit (Sigma-Aldrich, USA) for general cell membrane labelling was used, according to the manufacturer’s instructions, to label the lipids from the plasma membrane of the humans erythrocytes presented in *B. divergens* cultures. 1 × 10^7^ cells/ml from *B. divergens* cultures with ≈20% of parasitemia were washed with RPMI medium, resuspended in 250 µl of Diluent C and mixed with another 250 µl of Diluent C that contained 1 µl of PKH26. The sample was incubated at room temperature for 5 min and mixed with 500 µl of human serum for 1 min. Cells were centrifuged for 10 min at 400 × g and washed three times with complete medium. Then, cells were resuspended in 2 ml of complete medium and mitochondria from parasites were stained with MitoTracker Green FM (Thermo Fisher Scientific, OR, USA) at 500 nM. The sample was incubated for 45 min at 37 °C under growth conditions and centrifuged 2 min at 400 × g. The resulting pellet was resuspended in 2 ml of complete medium. Toxicity of PKH26 and MitoTracker green were determined together by incubating double-stained cells and non-stained cells in triplicate. All culture samples were placed in 6-well cell culture plates and incubated at 37 °C in a humidified atmosphere of 5% CO_2_ for 24 hours. Then, parasitemia progress from all samples was evaluated by Giemsa-staining and optical microscopy 24 hours later. Fluorescent samples were also observed immediately after staining and 19 and 24 hours later using a Leica TCS SP5 confocal laser microscope (Leica Microsystems, Germany) equipped with epifluorescence microscopy (Leica DMI 6000B microscope) and incubation systems to control temperature, humidity and CO_2_ conditions.

### Time-lapse recording and video processing

Stained cells (2 × 10^5^) were placed immediately after staining or 19 hours later in a glass-bottom Black 96-Well Plate, No. 1.5 Coverslip, 5 mm Glass Diameter, Uncoated (MatTek, MA, USA) and used for analysis by video microscopy. Cell count was performed using an automated cell counter (Tc10, Bio-Rad, Hercules, CA). Time-lapse video was conducted using a Leica TCS SP5 confocal laser microscope (Leica Microsystems). The 96-well plate was placed under the confocal microscope with 63x oil objective lens in a 5% CO_2_ environment at 37 °C. Time-lapse images of suspension cells were recorded at one frame per 0.753, 1.0 or 1.303 s. The videos generated by the Leica software (LAS AF) were exported as AVI documents and processed with ImageJ software (NIH) (http://rsh.info.nih.gov). The cell tracking were conducted using Imaris 9.1.2 software.

### Statistical analysis

The statistics was performed with GraphPad Prims 4.0 and IBM SPSS 22.0. The normality of each sample was assessed using the Kolmogorov-Smirnov and Shapiro-Wilk tests. When normal distribution was assumed, differences were estimated using ANOVA. For non-parametric distributions, the Kruskal-Wallis and Mann-Whitney tests were used. The Bonferroni test determined statistical significance. Probability values of 0.05 or less were considered statistically significant.

## Electronic supplementary material

Supplementary information

Supplementary Video S1

Supplementary Video S2

Supplementary Video S3

Supplementary Video S4

Supplementary Video S5

Supplementary Video S6

Supplementary Video S7

Supplementary Video S8

Supplementary Video S9

Supplementary Video S10

Supplementary Video S11

Supplementary Video S12

Supplementary Video S13

Supplementary Video S14

Supplementary Video S15

## Footnotes

1 Following Cursino *et al*.^6^, ‘stages’ are classifications of the parasite content of erythrocytes and are not necessarily sequential.
