# Supplementary material for: Kinetics of the invasion and egress processes of Babesia divergens, observed by time-lapse video microscopy
Source: Sci Rep. 2018 Sep 20;8:14116. doi: 10.1038/s41598-018-32349-7 (PMC6148197; doi:10.1038/s41598-018-32349-7)
Supplement: Supplementary file 1 — Supplementary information [file 41598_2018_32349_MOESM1_ESM.pdf]

Kinetics of the invasion and egress processes of *Babesiadivergens,* observed by time-lapse video microscopy

Authors

Elena Sevilla[1], Luis Miguel González[1], Daniel Luque[2], Jeremy Gray[3], and Estrella Montero[1,*]

Supplementary material

The following time-lapse videos show *B. divergens* parasites in green fluorescence and the erythrocyte plasma membrane in red fluorescence.

Supplementary Video S1. Time-lapse video microscopy captured the movements every 1 s of a free merozoite in the process of release together with three other merozoites from an infected erythrocyte. Outside the erythrocyte, the free merozoite moves vigorously at an average speed of 2.4 μm/s, reaching a maximum speed of 5.4 μm/s and a minimum of 0.03 μm/s, and covers a total distance of 123 μm in 52 seconds. The first contact of the merozoite with an erythrocyte takes place 6 s after exiting the cell. Then, the parasite recognizes new erythrocytes and establishes initial reversible contacts with several of them, causing a deep depression in the erythrocyte plasma membrane and dragging and flipping some of the erythrocytes during its trajectory.The merozoite is able to increase and decrease its speed throughout its trajectory while making contact with several erythrocytes without stopping its progress. The tracking of the parasite and the distance and speed measures were determined by Imaris 9.1.2. The time-lapsed between each frame is indicated in seconds.

Supplementary Video S2. Time-lapse video microscopy captured every 1s of three merozoites liberated from an infected erythrocyte. After merozoites are released from the infected erythrocyte and previous to the invasion process, it is possible to see in the red fluorescence channel, the plasmatic membrane of the infected erythrocyte retaining

the red PKH26 stain while the optical density of the intracellular cytoplasm decreases in the bright field channel (black-white color images). Then, one of the resulting free merozoites invades a new human erythrocyte. The video is showed on three individual channels together with the merged images. The time-lapsed between each frame is indicated in seconds.

Supplementary Video S3. Time-lapse video microscopy of the egress process of a paired pyriform captured every 0.753 s. The time-lapsed between each frame is indicated in seconds.

Supplementary Video S4. Time-lapse video microscopy of the egress process of a double paired pyriform captured every 0.753s. The time-lapsed between each frame is indicated in seconds.

Supplementary Video S5. Time-lapse video microscopy captured every 0.753 s of a *B. divergens* tetrad initiating a dramatic invagination and deformation of the infected erythrocyte during the egress process. The time-lapsed between each frame is indicated in seconds.

Supplementary Video S6. Time-lapse video microscopy captured every 0.753 s of a simultaneously egress of two merozoites from two different sides of the infected erythrocyte. The time-lapsed between each frame is indicated in seconds.

Supplementary Video S7. Time-lapse video microscopy captured every 1 s of a *B. divergens* merozoite from a tetrad, which initiates the egress process touching the erythrocyte plasma membrane at 26 s. Then the merozoite exits and the other merozoites follow. The time-lapsed between each frame is indicated in seconds.

Supplementary Video S8. Time-lapse video microscopy captured every 0.753 s of the egress process of a paired pyriform. The time-lapsed between each frame is indicated in seconds.

Supplementary Video S9. Time-lapse video microscopy captured every 1 s of the egress process of a double paired pyriform. Four merozoites are released from the infected erythrocyte. The last free merozoite is finally liberated after being trapped by the erythrocyte membrane during the process. The time-lapsed between each frame is indicated in seconds.

Supplementary Video S10. Time-lapse video microscopy captured every 1 s of multiple parasites within an infected erythrocyte. In this case, the invagination and deformation of the erythrocyte plasma membrane it is not possible to observe clearly. However, changes in the morphology of the infected erythrocyte, as a result of the egress process, are clearly visible. Thus, while the infected erythrocyte appears contracted and its diameter is reduced, several merozoites and one paired pyriform are being released from the infected erythrocytes at 6-22 s. One other paired pyriform remains within the erythrocyte. The time-lapsed between each frame is indicated in seconds.

Supplementary Video S11. Time-lapse video microscopy captured every 0.753 s of the egress process of an intact paired pyriform. The paired pyriform produces, from inside, a depression of the erythrocyte plasma membrane in 7.5 s. A few seconds later (≈4.5 s), while the infected erythrocyte appears contracted and its diameter is reduced, simultaneously the intact paired pyriform is released. The time-lapsed between each frame is indicated in seconds.

Supplementary Video S12. Time-lapse video microscopy captured every 1.303 s of the egress process of a double paired pyriform. The black arrow indicates two paired pyriforms within an infected erythrocyte. An invagination of the erythrocyte plasma membrane is visible in 183.7 s. After that, the diameter of the infected erythrocyte is reduced, while one resulting free merozoite from a detached paired pyriform is being

released from the cell alongside the other intact paired pyriform. The liberated paired pyriform, takes 47 s to detach into two free merozoites outside the infected erythrocyte. An unattached merozoite remains within the erythrocyte. The time-lapsed between each frame is indicated in seconds.

Supplementary Video S13. Time-lapse video microscopy captured every 1 s of two unattached pyriform parasites that move inside the cell previous to exit. An invagination of the erythrocyte plasma membrane is visible in 13-14 s. After that, the diameter of the infected erythrocyte is reduced and both merozoites exit the cell. The time-lapsed between each frame is indicated in seconds.

Supplementary Video S14. Schematic movie of the *B. divergens* invasion process.

Supplementary Video S15. Schematic movie of the *B. divergens* egress process.
